# Internal and External Root Resorption Management: A Report of Two Cases

**DOI:** 10.5005/jp-journals-10005-1186

**Published:** 2013-04-26

**Authors:** Nanditha Hegde, Mithra N Hegde

**Affiliations:** Assistant Professor, Father Muller Medical College, Mangalore Karnataka, India; Senior Professor and Head, Department of Conservative Dentistry and Endodontics, AB Shetty Memorial Institute of Dental Sciences Mangalore, Karnataka, India

**Keywords:** Inflammatory root resorption, Nonsurgical endodontic therapy, Mineral trioxide aggregate, Flowable gutta-percha system

## Abstract

The response of the dentoalveolar apparatus to infection is characterized by inflammation which may result in tooth resorption. Depending upon the type of resorption and etiology, different treatment regimens have been proposed. The following two cases demonstrate internal and external inflammatory root resorption arrest by conventional nonsurgical endodontic therapy combined with calcium hydroxide-iodoform dressing, mineral trioxide aggregate (MTA) and flowable gutta-percha system.The patient has been regularly recalled every 6 months and radiographically the apical lesion showed signs of healing and arrest of root resorption after 1 year and 6 months.

**How to cite this article:** Hegde N, Hegde MN. Internal and External Root Resorption Management: A Report of Two Cases. Int J Clin Pediatr Dent 2013;6(1):44-47.

## INTRODUCTION

Root resorption is of main concern to the endodontists. Dental clinicians can be faced with difficult diagnostic and treatment decisions with respect to tooth resorption.

Inflammatory resorption occurs when the predentin or precementum becomes mineralized, mechanically damaged or scraped off.^1,2^ Resorption is seen on the wall of the root canal (internal resorption) and on the external surface of the root (external resorption or cervical resorption) and it may be transient or progressive.^3^

Below are two interesting cases of extensive inflammatory root resorption occurring within a period of 1.5 year, including details of the history, examination, diagnosis and therapy.

## CASE REPORTS

### Case 1

A 15-year-old healthy boy was referred to the Department of Conservative Dentistry and Endodontics, AB Shetty Memorial Institute of Dental Sciences, Mangalore.

Patient had a chief complaint of fractured anterior composite restoration and tooth discoloration and his dentist had discovered a lesion in the apical region of the maxillary right central incisor.^11^ The patient had sustained trauma with respect to the maxillary central incisors 2 years back and had undergone endodontic treatment 1 month back with respect to 21 and 11.

**Fig. 1:**
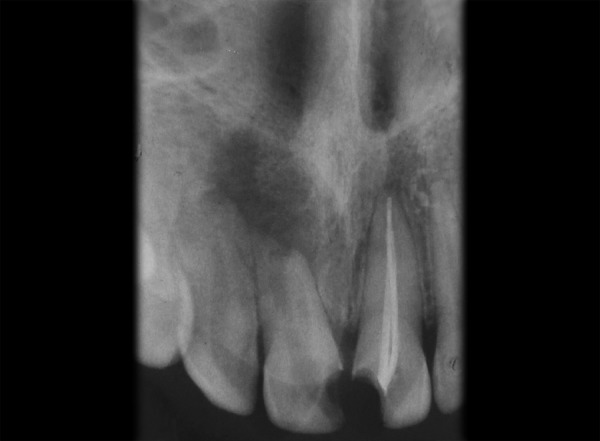
Maxillary right incisor of a 15-year-old male showing an infection induced communicating internal-external inflammatory root resorption and a related periapical inflammatory lesion in 11

Patient is moderately built and nourished and is afebrile at the time of examination. Clinical examination revealed – facial symmetry was normal, lips were competent. Endodontic testing found that the tooth was tender on percussion; vitality was negative and grade I mobility was present. Preoperative radiograph demonstrates widened canal system and radiolucent lesion in 11 (Fig. 1).

Based on the patient's history, and the clinical and radiographic examinations, the diagnosis was extensive inflammatory combined internal and external root resorption.^4^

When extensive inflammatory root resorption is diagnosed, there are generally three choices for treatment: (1) No treatment with eventual extraction when the tooth becomes symptomatic; (2) immediate extraction; (3) access, debridement and restoration of the resorptive lesion.^5^

### Treatment

**Fig. 2:**
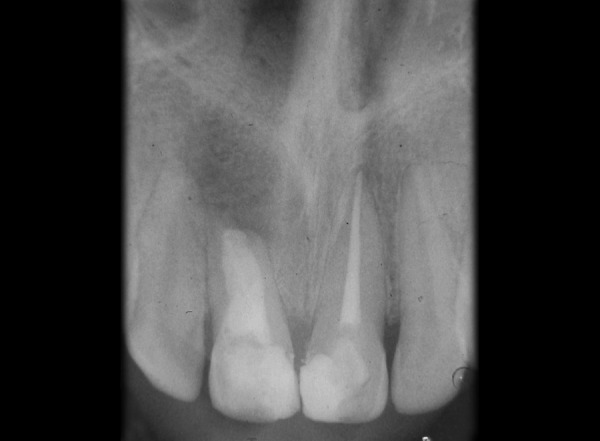
Radiograph at 9 months

**Fig. 3:**
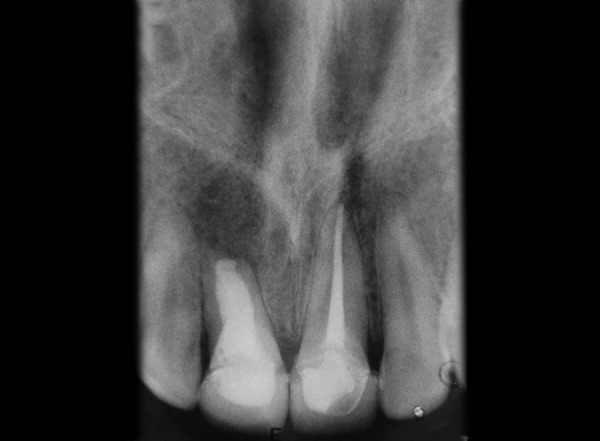
Radiograph at 1 year

**Fig. 4:**
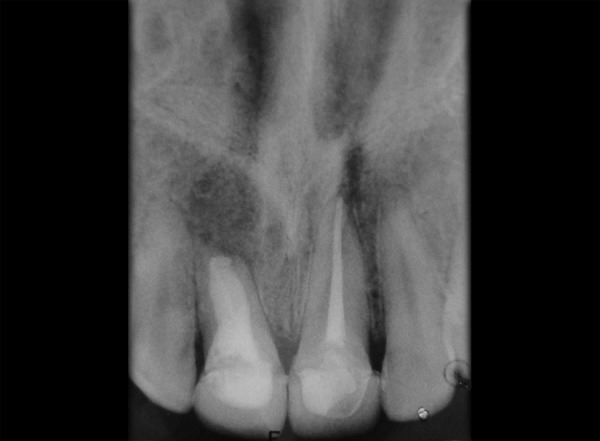
A 1-year and 6 months follow-up radiograph

Tooth 11, 21 was accessed and working length was determined (11 = 16 mm, 21 = 21.5 mm). Hemorrhage and exudate from the apical region of 11 was observed during the instrumentation. Microbrushes were used to scrub the calcium hydroxide paste in the lateral aspects of the root canal system. After 2 weeks calcium hydroxide was removed with a combination of hand NiTi files (Dentsply Maillefer; Ballaigues, Switzerland), sodium hypochlorite irrigation and EDTA (Glyde^TM^ File Prep, Dentsply Maillefer, Switzerland). Upon two more visits of calcium hydroxide dressing (Metapex, Meta Biomed Co. Ltd. Korea), obturation was initiated. Obturation was done with flowable cold filling gutta-percha system (Roeko GuttaFlow®2, Colténe/Whaledent GmbH + Co. KG, Germany) at 9-month recall (Fig. 2). At 12-month recall (Fig. 3) the intraoral periapical radiograph showed sufficient healing after which all ceramic crown was placed. A 1 year and 6 months recall (Fig. 4) showed patient being asymptomatic with radiograph showing no evidence of any breach or any periapical changes.

### Case 2

**Fig. 5:**
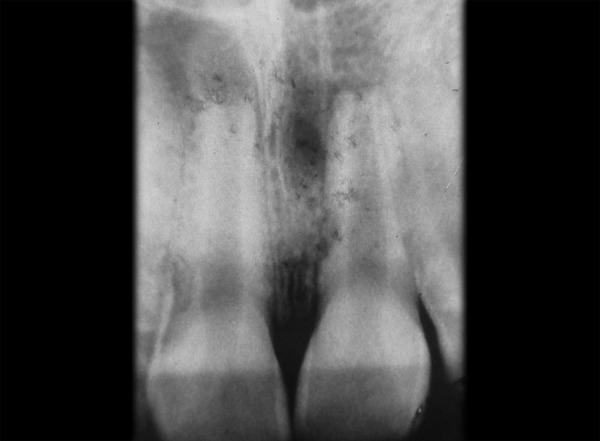
Maxillary central incisors of a 16-year-old female reveal signs of trauma induced external inflammatory root resorption in the right central incisor and apical resorption with periapical radiolucency with left central incisor

A 16-year-old girl had a chief complaint of pain in the maxillary anterior teeth with a history of trauma 3 years back. Extraoral signs and symptoms were similar to case 1. Endodontic testing found that the tooth was tender on percussion; vitality was negative. Preoperative radiograph demonstrates widened canal system and radiolucent apical lesion in 11 and 21, showing that signs of trauma induced external inflammatory replacement root resorption in 21 (arrows) and apical resorption in 11 (Fig. 5).

Based on the patient's history, and the clinical and radiographic examinations, the diagnosis was inflammatory combined internal and external replacement root resorption.^4^

### Treatment

**Fig. 6:**
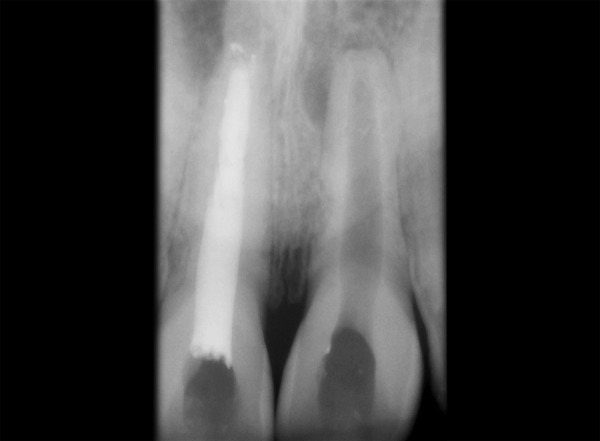
Radiographic appearance at 6 months

**Fig. 7:**
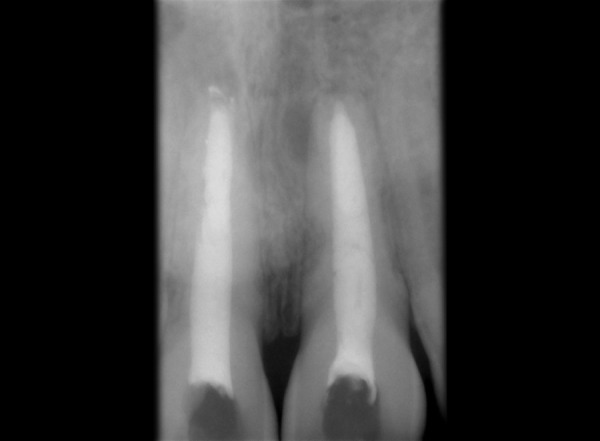
Radiographic appearance at 12 months

**Fig. 8:**
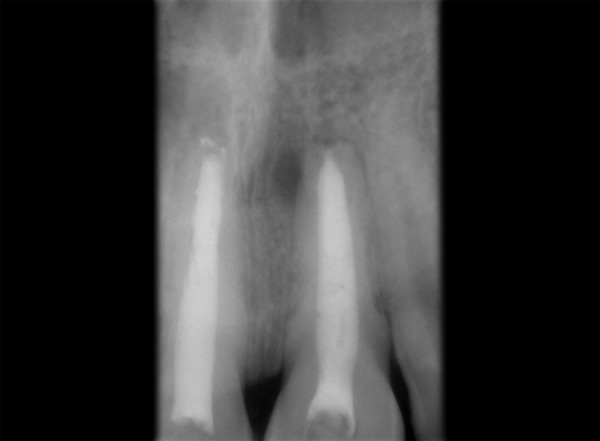
Radiographic appearance at 1 year 6 months

Tooth 11, 21 was accessed and working length was determined (11 = 20 mm, 21 = 21.5 mm). Hemorrhage and exudate from the apical region of 11 and 21 was observed. Biomechanical preparation was completed similar to the process described with case 1. Meneral trioxide aggregate (MTA) (Dentsply, Tulsa Dental, Johnson City, USA) was mixed and placed to form an apical stop. At 6-month recall, obturated with thermoplasticized gutta-percha (Calamus Dual 3D Obturating System, Dentsply, Maillefer) (Fig. 6). At 12-month recall (Fig. 7) the intraoral periapical radiograph showed sufficient healing of external root resorption in relation to 21 with replacement resorption and apical dome formation in 11. At 1 year and 6 months recall (Fig. 8) showed patient being asymptomatic with radiograph, showing no evidence of any periapical changes.

## DISCUSSION

Managing resorptive lesions can be challenging with unknown outcomes. Success depends on type of resorptive lesion (internal *vs* external resorption), location of lesion and size of the lesion.^6^ The most common cause of root resorption is trauma particularly in cases where the injury results in pulpal necrosis and damage to the root surface, leaving dentinal tubules exposed. Bacteria, bacterial byproducts and tissue breakdown products from within the root canal system stimulate inflammation in the adjacent periodontal tissues and lead to aggressive and progressive inflammatory resorption of the root.^6,7^

Treatment of root resorption is dependent on the etiology. In case where the resorption is due to pulpal necrosis and periodontal injury, nonsurgical pulp space therapy is performed. Complete chemomechanical preparation is considered as an essential step in root canal disinfection. However, total elimination of bacteria is difficult to accomplish. Intracanal medicament may help to eliminate surviving bacteria placed between appointments.^8^

Nonsurgical pulp space therapy combined with a calcium hydroxide dressing was recommended by Andreasen.^9,10^ MTA is also often used as repair material due to superior sealing ability, biocompatibility and fibroblastic stimulation.^11^ As an obturating material cold filling gutta-percha system (GuttaFlow®2) combines two products in one: Gutta-percha in powder form with a particle size of less than 30 μm and sealer.^12^ Good flow properties, low solubility and tight seal of the root canal due to its slight expansion, hence, no forces exerted on the weakened tooth structure as in comparison to thermomechanical or cold lateral compaction.

## CONCLUSION

Despite the serious damage to the root by external root resorption, nonsurgical pulp space therapy arrested the root resorption and regenerated the periapical tissue. Though the outcome cannot be predicted, it is worth an effort to try slow down the resorption process and maintain the tooth as long as possible in the arch for esthetics, mastication and natural space maintenance and above all psychological uplift of the young minds.
